# Book Reviews

**Published:** 1807-05

**Authors:** 


### CRITICAL ANALYSIS
### OF THE
### RECENT PUBLICATIONS
### ON THE
### DIFFERENT BRANCHES OF PHYSIC, SURGERY, AND MEDICAL PHILOSOPHY.

*The Edinburgh Journal, No.* 10.

" Article I.—*Observations on the Amputation of the Hip Joint.* By James Veitch, Esq. Surgeon, Member of the Royal Medical and Physical Societies of Edinburgh, and lately Surgeon of the Royal Hospital, Plymouth.

An operation to which strong nerves, an accurate eye, and habitual delicacy of managing his instruments and fingers, could never ver reconcile Mr. Pott, as here recommended by practical success. As these are questions which can only be decided by the teachers of Anatomy, we shall never engage in them. It is easy to object, but no one, who has not very large opportunities of practical knowledge, can be equal to all the difficulties which may occur in so complicated, and, as it has sometimes been called, so butcherly an operation.

In the course of the above paper Mr. Veitch acknowledges himself the author of those remarks on ligatures, &c. published with the signature of J. D. Article "The Enquirer," in a former Edinburgh Journal.

We before expressed our dissatisfaction at the manner in which the author had availed himself of Dr. Jones's discoveries, without any thing more than a casual reference to that meritorious experimentor and candid writer.

Article 2.—*Letter to Dr. James Hamilton, Senior Physician to the Royal Infirmary of Edinburgh, on the Cure of Scarlatina.* By JONATHAN BINNS, Physician, Lancaster. Communicated by Dr. Hamilton.

This is a tedious paper on a subject which is almost worn out. We mean not to say that Scarlatina is not often a serious complaint; but, as Sydenham observes of small-pox, in many instances bad treatment will not kill, and in others no remedies will cure. On this account all well digested practical remarks are valuable; but, after all, the practitioner must act for himself, as no two serious cases will be in all respects similar.

Article 3.—*Observations on the Advantages derived from the free Administration of Purgatives.* By CHARLES MORGAN, M. B. of Dover.

THIS paper contains a few useful remarks on the importance of attending to the alvine excretions of our patients. We know not those southern practitioners, who are so much afraid of purgatives, or even of cold affusion; but we are forced to admit that these, and many other remedies, have not always succeeded in the south,* with that certainty and uniformity which some of the northern writers have taught us to expect.

Article 4.—*Some Account of the Baths of Leuk, in the Valais.* By HENRY REEVE, M. D. F. L. S.

Article 5.—*Case of Small-Pox occurring in the Fœtus.* By JAMES LAIRD, M. D. Physician to the Cary Street Dispensary.

THIS paper contains two facts worthy of record, though neither of them new. A woman was seized with small-pox in the fifth month

* See Dr. Willan's Diseases of London, p. 235.

month of her pregnancy. The child seems, by the dates, and its appearance after birth, to have died about the turn of the disease in itself. The author remarks, that it is difficult to say at what period of utero-gestation the embryo becomes liable to suffer from the diseased actions of the parent. From what has been observed, he adds, of the syphilitic virus we are at liberty to conclude that this susceptibily is very early established." We should be glad to be informed of the observations which establish the law relative to the early susceptibility to syphilis, as our own reading, memory, or practice, are insufficient to furnish any thing conclusive on the subject.

The other fact, with which this paper concludes, is an instance of a second occurrence of casual small-pox, in the same person, at the distance of fourteen years.

Article 6.—*Observations on Cow-Pox.* By John Burns, Teacher of Midwifery, Glasgow.

This paper contains another case of Small-pox occurring after Cow-pox. The author concludes with some enquiries as to the mode in which we can ascertain the security of our patients, and seems disposed to admit Mr. Bryce's plan of a re-inoculation about the sixth or seventh day. We have often been surprised at the security expected from this practice. Mr. Burns informs us that his patient went through the regular process of vaccination, which he describes with sufficient accuracy. Can we therefore suppose, if she had been vaccinated a second time, on one of the days proposed, that the second insertion would not have followed the usual process, which has constantly been observed on these occasions.

Article 7.—*On the common Cause of the Puriform Ophthalmia in New-born Children.* By Benjamin Gibson, Esq. Surgeon to the Manchester Infirmary.

Mr. Gibson suspects, with much reason, that this disease arises from some impression received by the child during its passage thro' the vagina, in consequence of the secretion of the latter. He proposes, as a prevention, first, to cure, if possible, fluor albus in the mother, should she be afflicted during pregnancy. Secondly, if that cannot be done, to remove as much of the discharge as possible from the vagina at the time of delivery. Thirdly, to pay, at all events, particular attention to the eyes of the child, by washing them immediately after delivery with a liquid calculated to remove the offending matter, or to prevent its noxious effects. The last is, we think, as much as can be attempted with any advantage; at the same time, we are ready to thank the Author for the useful hints suggested in his paper.

Article 8.—*Letter to Professor Loder, of Jena, regarding the Operation of spurious Aneurism.* By Professor Scarpa, of Pavia.

We have before now remarked, that the present difficulty of procuring curing foreign Journals, makes them fair subjects for transcribing into British publication; but we must again repeat, that selections should never be admitted unless they offer something new. The above cases, though they do credit to Professor Scarpa's skill in operation, afford nothing new to the English surgeon.

Article 9.—*Concise Observations on Anæmia, a Disease which attacked all the Workmen of a Gallery in a Coal Mine, now worked at Aurain Francs and Vieux Conde, near Valenciennes, and which has been observed and treated in four of these Workmen at the Hospital of the School of Medicine at Paris.*

This paper is also a selection from Corvissart's Journal; it is, however, much more worthy to be transcribed than the former. Our readers will perceive, from the derivation of the name of this disease, ab ἀ privativo et αἷμα, that it must be very similar in its appearance to the worst kind of chlorosis. It might be said to resemble still more the effects of lead, excepting that we have no account of paralytic joints, and that palpitations of the heart are a common attendant on chlorosis. The following may serve as a sufficient description to characterize the disease, should it occur to practitioners who have never before seen it. To give every leading information, we prefix the account of the coal pit in which the four patients had worked who were sent to the Medical School at Paris.

"This gallery, however, differs in no respect from the rest; it is of the same depth, being 234 metres (120 fathoms) below ground; it is excavated in the same manner, only, it is longer, and less easily admits of a renewal of air; and the apertures that have been made for this purpose have not had any beneficial effect. Its temperature is 64° Fahr.; it exhales an odour of sulphurated hydrogen gas; in it respiration is difficult; and the workmen affirm, that the water which filters across the mine, on touching their hands, or the naked parts of their bodies, produces blisters and boils. Nevertheless, they have had the imprudence, notwithstanding the fresh water which is provided, and the prohibitions which have been given, to use it sometimes to allay their thirst; and yet, before the summer of the eleventh year, no disease had been observed similar to that which I am now going to describe.

"According to the description of it sent to the School of Medicine, it chiefly has the following characters: It commences with violent colics, pains in the intestines and stomach, difficulty in respiration, palpitations, loss of strength, inflamation of the belly, and black and green stools. This state continues for 10 or 12 days, or more; then the abdominal pains cease; the pulse remains feeble, contracted, and weak; the skin loses its colour, and gets a yellow tinge; walking is difficult, and accompanied by extreme fatigue; frequent palpitations cause an extremely painful state of anxiety; the face is swollen; and there are frequent, and even habitual sweats. This state continues for many months, and even even more than a year, and is always attended by wasting and emaciation. At last, the original symptoms recur, with violent headachs, frequent faintings, intolerance of light and sound, inflation and pain of the belly, and purulent stools. These last torments are soon terminated by death.

"When these details were transmitted to the Society of the School of Medicine, out of fifty attacked with it, three had died, and none were cured. Bark, camphor, opium, wine, spare diet, and many other means which seemed proper, were used, without success.

"The society upon deliberation, drew out at first a plan of treatment, conformable to the general indications, in which it considered particularly the analogy which the disease seemed to present with the metallic colics, and with some chronic consequences of the *asphyxia*, known to vintagers under the name of *plomb*. Then, upon the supposition, unfortunately too probable, of the inutility of a plan of treatment drawn up in a general way, from apparent indications, the Society proposed, according to particular observations, and in an empirical manner, the trial of mercurial frictions, the internal use of oxygenated muriatic acid, diluted with water, &c. But, above all, it demanded more particular details of the state of the places; recommended the opening of the dead bodies, which had hitherto been omitted; and expressed a desire that it should have an opportunity of directly observing some of those affected, which could be easily done, since the second stage of the disease was sufficiently long to enable some of the workmen, most severely affected, to be sent to Paris, which has accordingly been done.

"Accordingly, four pitmen were sent to Paris, with the necessary information, either written, or delivered verbally by the physician who attended them. A plan of the galleries of the mine, air and water taken in the infected gallery, farther details concerning the sick, and minute accounts of the examination of two dead bodies were given. It was observed, that the methods proposed by the Society of the School of Medicine had been comparatively employed; that of six workmen to whom the frictions had been applied, two were cured, and had resumed their labour, but the other four were yet under treatment; and that doubts were entertained, whether to this remedy they were indebted for the cure. In fine, it was observed, that among the infected workmen, the number of whom was increasing, there were two states, considerably different: Some, having been attacked in the mine itself, were very severely affected, and, of these, none had been cured; but others had been seized with the disease since the gallery had been shut up, and to many of these health had been restored.

"We received no written account relative to the particular history of the men who have been sent; but as they had been ill for eight, twelve, or fifteen months, and as they began to be affected when they were at work, we believe that they ought to be ranked in the first class.

"When they arrived, they were of a wan yellow colour, not like that of men affected by the jaundice, but like white wax which has been long kept. They were œdematous; the face, as also the superior extremities, were swollen in a particular manner; the legs were also swelled, but this perhaps might be owing to the fatigue of the journey; and indeed, after two days rest, they became less œdematous, and as lean as in their natural state. The loss of colour was general over the surface of the body; and not only was the skin wan and yellowish, but the conjunctiva, the inside of the eye-lids, the lips and mouth, and the tongue, were also deprived of their natural colour. No ramification of capillary vessels appeared on any of these; and, in general, no vein was visible on the arm, bend of the fore-arm, and back of the hand, either by its colour or convexity.

"The belly presented no disorder sensible to the touch, only the mesentery seemed to be considerably larger, though soft; and the hypochondriac regions appeared to be free.

"The pulse was constantly quick, beating eighty, ninety, and a hundred times in a minute, without, however, the skin being hot; yet, at any time, when actual fever supervened, the skin became warm, the pulse was also quickened, and the other symptoms announced a change in the habitual action of the functions. Besides, the pulse was frequently altered by the palpitations; and the heart, even when it did not palpitate, beat very strongly against the ribs.

"Another symptom, no less constant, but different from palpitations, and which may well be supposed to be a consequence of them, was the patients inability to walk, without being obliged to stop, and to sit down at the end of a few steps. They could not even mount one pair of stairs, without being obliged to sit down frequently on the staircase. Still the percussion of the breast did not indicate any congestion in the viscera of that cavity, nor any quantity of diffused water. The palms of their hands were frequently moist, and they had habitual night-sweats. Besides, these men, one of them excepted, had a good appetite; they ate with avidity, food which was agreeable to their taste. They disliked butcher's meat; and had other tastes, which appeared to be the effect of habit, rather than of disease. They digested easily; but their stools did not indicate a perfect or equal digestion. They were often half liquid, sometimes liquid, brown, yellow, and sometimes green. The urine, in general, had its ordinary colour.

"Thus the characteristic symptoms were the universal loss of colour and yellow tinge of the skin, swelling, impossibility of walking without suffocation, palpitations, and habitual sweats. Some days after the arrival of these men, and when they were familiarized with the objects around them, considerable attention was paid to the regulation of their diet. Substantial aliments and foods, such as they desired, and for the most part roasted, were given them. They were also supplied, at first, with excellent beer, then with with good wine, and bread well baked. Their appetites and desires were satisfied, as far as prudence would permit, consistently with the necessity of selecting their food in a proper manner. In a word, nothing was neglected to prevent any moral affection from aggravating their disease.

"Guided by the report of the physician who accompanied them, and the presumed success of the mercurial frictions, they were administered, at first every third day, in doses of a drachm, and, at the same time, antiscorbutic wine was given them, and bitter drink, made of hops and little centaury; which corresponded both to the apparent indication drawn from the symptoms and the inconvenience naturally to be dreaded from the use of the mercury.

"During this treatment, one of the patients fell a victim to the disease. He had always appeared more languid, and had less appetite than the rest, and was affected with a rheum for a few days after his arrival. The frictions had been commenced upon him, as well as the rest, on the 15th of *Messidor*, but were discontinued on the 25th, because a very sensible tendency to fever was observed during these days. The fever continued; and on the 6th of *Thermidor*, it assumed a serious aspect; it was continued in its form, with pain in the limbs, and acute head-ach, and the pulse was even hard. Notwithstanding the violence of the fever, and the dryness and burning of the skin, neither the tongue, the lips, nor conjunctiva was coloured; and the tongue was perfectly clean, as well as that of the other patients. The belly was swollen, and painful to the touch; and along the inferior edge of the ribs of the right side, a resistance was felt, which was supposed to be the liver. At the end of 48 hours, the fever abated, the pulse became weak, and there were efforts of vomiting, which only produced some slimy matter, to assist which, an emetic cordial potion was given, without the desired success. Great oppression, a feeble and intermitting pulse, and cold extremities, succeeded. A blister applied to the side, and repeated as a stimulant, raised the pulse only for some hours. The intermission increased, as well as the cold extremities and oppression of the breast, and the patient at last died.

"The belly was a little swollen; there were no livid marks on the skin, and the colour was in general the same as in the course of the disease.

"The abdomen contained no serous exudation. The intestines, and especially the colon, were very much distended; and the fat, both subcutaneous, and in the omentum and mesentery, was very yellow. The liver was small, and did not project beyond the ribs; it was soft and pliable in every part; it was of a pale yellow colour, both externally, and in its substance, which was soft and unctuous to the touch. The gall bladder was half full of bile, of a colour like the yolk of an egg; and when analysed, was found to contain much coagulable albumen, and posessed some other

 peculiar peculiar properties. The spleen was small, and softer than ordinary; and the liquid which flowed from it, as it generally is, was red, like the dregs of red wine.

"The stomach, when opened, was found half full of a liquid, coloured like the dregs of wine. The duodenum and the jejunum were lined with a mucous membrane, of a similar colour; and when that mucous was removed, the membrane, both in the stomach and intestines, in all their extent, appeared white and sound. The matter contained in the rectum was thick and figured, and of a greenish brown colour. All the other abdominal viscera were sound.

"In the cavity of the thorax, the right lung adhered almost every where to the pleura, and especially on its anterior part; but the left lung was almost entirely free. In neither was there any remarkable quantity of serosity; both were light, decrepitated under the fingers, and there was no congestion. They were externally white, and mottled with dark blue spots; and, on incision, a frothy yellowish serosity issued from all points of their substance, but not from any preternatural collection. The heart was of a very ordinary size, and its flesh as pale as that of muscles which have been washed and macerated. Its parietes were soft, and the *columnæ carneæ* small. Its structure was not at all affected. Not a drop of red blood escaped from any of its cavities; but in the left ventricle, a coagulum, as pale as the flesh of the heart itself, was observed, which contained no perceptible portion of colouring matter; the pericardium contained no serosity.

"The brain was white; the cineritious substance pale, and little distinguished from the medullary substance. Two or three scruples of serosity only were found in the posterior part of the left ventricle; and the chcroid plexus was red, but very pale.

"In the three cavities, all the vessels, both arteries and veins, were destitute of coloured blood, and contained only a small quantity of a serous liquid. No blood was found in the aorta as far as its crural subdivisions, nor in the axillaries as far as the brachial subdivisions, nor in the accompanying veins, nor in the system of the hepatic vessels, nor in any of the sinuses of the brain. Upon making a deep incision into the flesh of the thighs, there flowed out a small quantity of liquid and black blood, but from no other part did any flow. The flesh of the muscles which cover the thorax was exceedingly red; but that of the extremities not so much so.

"It is worthy of remark, that the same absence of blood occurred in all the dissections made where the disease appeared; that it agrees with the general loss of colour observed in all the parts which are naturally red, and especially the surface, where the red blood is obviously admitted into the capillary system. It may be regarded, therefore, as a state particularly dependent on the disease; as exhibiting itself by evident symptoms during its whole whole continuance; and arriving at its height when the disease is at its termination, or its last stage."

From the well known properties of iron in inducing oxygenation of the blood, iron filings were given in the proportion of a drachm per day. Though convalescence was very tedious, and in some not completed when the account was given, yet the prospect was sufficient to justify the perseverance in, and the trusting almost exclusively to, that remedy.

We have given this long extract as a specimen of French accuracy, in describing diseases. When we reflect on the superiority of M. Valentine's account of the American fever, compared with what we can boast from most of our own practitioners, it obliges us to admit, that how deficient soever the French physicians may be in physiology, we should do well to copy them in their accurate mode of tracing symptoms and describing appearances.

The Number concludes with a very entertaining and by no means useless paper on the cure of scrophula by the royal touch. It affords a curious instance of the credulity of the human mind, which on this occasion extended to the best informed among the Faculty. Of the latter, however, we must recollect that the writers were *Royal Surgeons.*

" John Browne, Surgeon to King Charles II. appears to have been a man of established character, and high reputation in his profession; his book is approved by the President and Fellows of the College of Physicians, and the most eminent surgeons of his day. He seems to have been warmly attached to his sovereign, as he suffers no opportunity to escape without saying something in favour of the divine right of kings; and he is violent in his outcry against the dissenters and disloyal subjects; " with any of the canting tribe (he says), I hope this treatise will never get favour." During the two years of his exile, the King used to touch at different places where he was staying, and Browne relates several remarkable instances of cures performed by him during the troubles, as well as after the restoration; and he confidently asserts, that the usurper *Cromwell* tried in vain to exercise this royal prerogative, " he having no more right to the healing power than he had to the regal jurisdiction." There was a Scotch merchant, who made it his business, every spring and fall, to bring people from Scotland and Newcastle, troubled with the evil, to the king, wherever he was, during the political troubles of his kingdom, as at Brussels, Breda, Bruges, Antwerp, and the like; and, before his return, he acquainted the surgeon in ordinary how those persons were which he carried back with him, after being touched. The most interesting part of Browne's work is the copy of the register, shewing the number of persons in each month, for a period of 21 years, I shall select the two years distinguished for the largest and the smallest number of persons whose cases are

 registered registered, from "An account of persons touched by his most sacred Majesty King Charles the Second, for the cure of the king's-evil, from May 1667 to May 1682, taken from a book or register thereof, kept by Mr Thomas Donkley, keeper of his Majesties closet, belonging to his Majesties royal chapel."

| 1669 | | 1682 | |
|---|---|---|---|
| *Months* | *No. of Persons.* | *Months.* | *No. of Persons.* |
| May - - | 178 | May - - | 260 |
| June - - | 31 | June - - | 339 |
| July - - | 40 | July - - | 106 |
| August - - | 13 | August - - | 49 |
| September - | 53 | September - | 1027 |
| October - | — | October - | 221 |
| November - | 512 | November - | 1371 |
| December - | 205 | December - | 815 |
| January - | — | January - - | 4 |
| February - | 131 | February - | 1594 |
| March - - | 819 | March - - | 220 |
| April - - | 1001 | April - - | 2471 |
| | 2983 | | 8577 |

"Notwithstanding the numbers who had been touched and healed by his sacred Majesty after his restoration, were so great, as to amount to a considerable proportion of the whole nation, yet upon any new declaration of a fresh healing, they were again as fast, as if no one had ever applied before. "A thing (says Browne) as monstrous as strange!" This proves, that the touch did not always succeed; it could not at any rate prevent a relapse; and it is confessed, "that although it did not always answer expectation, yet its effects were wonderful, and its cure most frequent, as is, and hath been sufficiently and satisfactorily demonstrated. To add any more evidence to what has been already adduced, is almost unnecessary, yet I cannot withhold the testimony of *Wiseman*, an author of credit, and of a standard work in surgery. One of his surgical treatises is written expressly upon the king's-evil, and he there speaks of the royal touch in the following strong terms: "I myself have been a frequent eye-witness of many hundreds of cures performed by his Majesty's touch alone, without any assistance of chirurgery; and those, many of them such as had tired out the endeavours of able chirurgeons before they came thither. It were endless to recite what I myself have seen, and what I have received acknowledgements of by letter, not only from the several parts of this nation, but also from Ireland, Scotland, Jersey, and Germany."— Wiseman's Surgery.

This entertaining little extract will teach us not to wonder at the accounts we have read of the virtues of particular plants, when merely suspended from the neck, or at the success of a Manuduc, a Prescott,

Prescot, or, as we have lately read, of the virtues expected from the touch of a dead criminal.

---

*History and Analysis of the Mineral Waters, situated at Butterby, near Durham.* By W. R. Clanny, M. D. Honorary Member of the Royal Philosophical Society, Edinburgh; Physician to the Durham Infirmary, &c.

The importance of mineral waters becomes daily better understood, and the probability that chemistry cannot detect, nor consequently imitate all the properties contained in some of them.— The present useful little performance exhibits a lively description of Durham, and the source of the waters, an analysis of their properties, account of their medical effects, and of the diseases in which they have been found servicable.

---

*An Address to the Professors of Physic and Surgery of London and Westminster, proposing the Institution of a Society for investigating the Cause, Symptoms and Cure of the Hydrophobia.* 8vo.

Nothing can be better intended than the proposal of this Author; we only regret that his modesty has induced him to conceal his name, and to leave the completion of his plan to others. Had he given a full statement of his designs, fixed the time and place for a first meeting, and added his name to such an advertisement, we doubt not that a beginning would have been made of his laudable undertaking, and in the end that mankind would be profited by it.

---

*An Account of the Ophthalmia which has appeared in England since the Return of the British Army from Egypt.* By John Vetch, M. D. Member of the Med. Soc. of Edinburgh, and Assistant Surgeon to the 54th Foot. 8vo.

We shall pass over the Preface, in which the Author touches on the same ground as Dr. Edmonston, relative to the account given of this disease by the ancients. The coincidence is certainly curious, but as in many other cases the descriptions are too loose or too obscure, to ascertain the precise history, and sometimes even the character of the disease.

A much more important consideration is, the very extensive field of practice which the state of the regiment afforded, and the consequent variety of symptoms which occurred; the inefficacy of every mode of practice, till one was determined upon as bold as the necessity of decision became imperious, and not less rational, whether we consider the severity of the symptoms, or the age, habits and characters of the patients. We shall also avoid dwelling on the description of the symptoms, because their variety shows that they differ from what has been before remarked in scarcely any thing excepting the greater severity in some and the conclusive proofs of the contagious quality in all.

Ther

There are, however, two remarks in our Author, which are not so well related in any other writer. The first is, that whilst some companies were quartered in Rumney Marsh, the disease seemed to partake of the intermittent form; the second is, the accuracy with which he describes the bursting of the cornea, all the antecedent and attendant symptoms and its consequences. But we have reserved our transcript for the mode of cure, not only on account of its novelty and the marked success with which it was attended, but because, as we observed in our remarks on Drs. Jackson and Sutton, we conceive the present race of practitioners have been, for the most part, much too sparing of the lancet.

We are first informed, that after every remedy, topical and general, had been tried, which were found in books, or suggested by experience —

"The urgent nature of the disease, and the inefficacy of the previous treatment, demanded the most active measures; but at the same time it required much professional knowledge to guide them. Notwithstanding the undisturbed state of the system, and apparently in opposition to the experience which had been already gained, Mr. Knight proposed the most decided antiphlogistic regimen, with the regular exhibition of purgatives, and above all, the use of the lancet, with a freedom far beyond what had been formerly thought of.

"The decided advantage which has resulted from venæsection, when carried to a proper extent, sufficiently shews the cause of its former inefficacy. I must own, that after having examined the disease, I could with difficulty, at first, reconcile my principles to this practice, but the success was beyond all dispute. I have now seen it carried farther than I could have dared to suppose, and in every instance has its efficacy corresponded with the extent. When this rigorous system of depletion was first commenced, it was generally employed in those cases where the violence of the disease threatened the rupture of the cornea. In those, it was resorted to on the accession of the pain, which it sometimes relieved, without seeming to prevent its recurrence, at others no sensible benefit was derived. Under such circumstances, it was often repeated to the eighth or ninth time, without the decided benefit which followed its more extensive employment. I am even inclined to think, that the relief which was attributed to the venesæction was, as it was then performed, the effect of the natural termination of the paroxysm. But allowing that it produced this temporary relief, the benefit which resulted from checking in succession a number of paroxysms, seemed hardly to compensate for the expense at which it was obtained. As yet we had no certain rules for regulating the quantity of blood — seldom more than ℥xxx were taken at a time — and it was chiefly had recourse to during the paroxysm. The bleeding was afterwards adopted more fully in the early stage of the disease, and the quantity taken away was only measured by the effects it produced on the system; when all doubts as to the propriety propriety of the practice ceased, from the benefit which immediately attended it. The disease seems now to be more under our controul, than any other in which the same remedy is employed. A few cases will still occur, in which it extends to the internal points of the eye; but these instances are rare, and merely shew the violence with which it would occur, if opposed by less vigorous measures. In every case, where on the first appearance of the disease, bleeding is employed to the proper extent, its effects are no less perceptible to the patient than to the surgeon. The diminished vascularity is the first effect which ensues, and before the end of the operation, the eye will often become nearly of its natural appearance. The cessation of all uneasiness should be the *sine qua non* of stopping the flow of blood. This, in a robust man, will often not be obtained until thirty or forty ounces have been taken away, and in a few, *deliquium* will take place before it is effected; one or other of these effects should always be procured. By the repetition of this remedy, on every aggravation of the disease, whether in the appearance of the eye, or in the sensations of the patient, we may, in ninety cases in the hundred, prevent it from arriving at the second stage. If however, in defiance of this treatment, it assumes its violent form, the same means are, if possible, to be more fully persevered in. In this stage, the patient will often have bleeding to a greater extent; in many, fifty or sixty ounces must be taken away to relieve the pain, or bring on syncope: but we can always rely with certainty on the benefit which will ensue when either of these effects is produced. In every case where such practice is employed, however violent the tendency of the disease may be, its fatal termination will infallibly be prevented, and with much less expense to the patient than by smaller and more frequent bleeding. According to the rules above laid down for the extent to which this evacuation is to be carried, the quantity taken away must be in proportion to the strength of the patient, hence few cases will occur where the practice cannot be put in execution.

"With the same confidence with which this extensive venæsection is recommended, I would dissuade from a less free use of the lancet. In the former case, it appears to operate not so much by lessening the disposition to the disease, as the power of the constitution to carry it on; in the latter, it debilitates the patient, without producing either of these effects. In every case where the patient was most willing to have the operation repeated, it had formerly been carried to the greatest extent. The considerable time before the practice was so generally reconciled to our minds, was in consequence of its too sparing adoption, and should serve as a seasonable caution to the practice of others; and in adopting the practice, the first rule is to overlook every thing except the disease. The blood was sometimes taken from the jugular vein and temporal arteries, without any superior advantage. From the great ease with which the quantity of blood can always be obtained from from the veins of the arm, in a given time they were generally resorted to.

"In a former part of this Treatise, it was mentioned that changes in the pulse were more frequent after the adoption of the treatment now related. These changes were seldom the immediate result of venæsection; and the only alteration which can be accurately ascribed to it, was a greater degree of compressibility, and being more affected by the posture of the patient. The appearance of the blood was often no less at variance with the practice than the general state of the system. In some cases, where venæsection had been more frequently employed, in consequence of its effects having been productive of less good, the blood remained cupped and buffy, until, from the debility of the patient, or from the inefficacy of the treatment, the operation could be no longer repeated. Such instances were, however, extremely rare: in those, the quantity taken had not been sufficiently great. In the greatest number of cases, where bleeding was most extensively employed, no such cupped or buffy appearance took place.

"The alteration which this treatment produced in the symptoms of the second stage was very remarkable, nearly the same degree of tumefaction took place both in the palpebræ and the conjunctiva, where it covers the eye; the former, however, were no longer subject to the same degree of eversion, the quantity of pus was much diminished, and in no case did granulation become a troublesome symptom."——

We have given a larger extract than our limits will usually permit from a work of this size, not only on account of the success which attended this bold practice, but because, as we before remarked, it seems absolutely necessary to convince the practitioners of the present day, that where we expect relief from the lancet, we must be governed in its use not by the pulse or the quantity of blood taken, but by the effects on the disease. Of this, in acute cases, we can form only a precarious opinion, unless our practice is decisive. This is confirmed, not only by the writers we have before mentioned, but by many others; among the rest, by Doctor Pinkard, who compares the cautious bleedings of some practitioners in the yellow fever to an attempt at extinguishing a volcano as we would extinguish a culinary fire.

